# Benchmarking Drug Regulatory Systems for Capacity Building: An Integrative Review of Tools, Practice, and Recommendations

**DOI:** 10.34172/ijhpm.2023.8100

**Published:** 2023-10-16

**Authors:** Junnan Shi, Xianwen Chen, Hao Hu, Carolina Oi Lam Ung

**Affiliations:** ^1^State Key Laboratory of Quality Research in Chinese Medicine, Institute of Chinese Medical Sciences, University of Macau, Macao SAR, China.; ^2^Centre for Pharmaceutical Regulatory Sciences, University of Macau, Macao SAR, China.; ^3^Department of Public Health and Medicinal Administration, Faculty of Health Sciences, University of Macau, Macao SAR, China.

**Keywords:** Regulatory Capacity, Capacity Building, Global Benchmarking Tool, Drug Regulatory Systems, National Regulatory Authorities

## Abstract

**Background:** Benchmarking has been increasingly used on drug regulatory systems to achieve sustainable pharmaceutical system strengthening. This study aimed to identify the scope, tools and benefits of benchmarking regulatory capacities and the most recent development in such phenomenon.

**Methods:** This study employed an integrative and critical review of the literature and documents on benchmarking drug regulatory capacities identified from 6 databases and 5 websites of related organizations and government agencies in compliance with the Preferred Reporting Items for Systematic Review (PRISMA) guidelines.

**Results:** Forty-three studies and 6 documents about regulatory benchmarking published between 2005 and 2022 were included in this review. Five benchmarking assessment tools or programmes recommended or adopted by international organizations or government agencies had been identified, which collectively covered 12 major regulatory functions (4 at system level and 8 at operational level) involving 9 indicator categories and 382 sub-indicators. Benchmarking drug regulatory systems was reportedly employed at national, regional and international levels for either internal assessment (mostly on regulatory system establishment, drug review process and post marketing surveillance) or external evaluation (mostly on regulatory standards, drug review process and pharmacovigilance systems) to assess current status, monitor performance, determine major challenges and inform actions for capacity building. Priority of actions in areas such as regulatory process, resources allocation, cooperation and communication, and stakeholder engagement have been suggested for strengthening drug regulatory systems. Nevertheless, the evidence about benchmarking in optimizing regulatory capacities remained underreported.

**Conclusion:** This integrative review depicted a framework for decision-makers about why and how benchmarking drug regulatory systems should be undertaken. For effective benchmarking, well-informed decisions about the goals, the scope, the choice of reference points and benchmarking tools are essential to guide the implementation strategies. Further studies about the positive effects of regulatory benchmarking are warranted to engage continuous commitment to the practice.

## Background

 Benchmarking, a common systematic practice that allows organizations to measure and compare key practice metrics to understand what, how and where changes are needed to improve performance, has been increasing employed on drug regulatory systems to achieve pharmaceutical system strengthening and universal health coverage.^1^ Drug regulatory systems operated by national regulatory authorities (NRAs) play an integral role in the pharmaceutical system destined to ensure equitable access to essential medical products, vaccines and technologies of assured quality, safety, efficacy and cost-effectiveness, and their scientifically sound and cost-effective use.^2^ The quest to excel the regulatory practice is further heightened amid the challenges brought about by, among other forces, the innovation and technology advancement, and major public health incidents.

 By comparing their performance and capacities against a reference point, NRAs can determine how they perform, their weaknesses and strengths, and how to prioritise actions to continuously improve quality use of pharmaceutical products with respect to the local context.^3^ It is envisioned that benchmarking NRAs would also benefit the development of strategies for promoting regulatory practice standardization for transnational harmonization, reliance and recognition, as well as system resilience at country level in response to globalization of pharmaceutical products.^4,5^

 Drawing on the integrated approach of benchmarking and the experiences of public sector benchmarking,^6-9^ the practices of benchmarking drug regulatory capacities are complicated. First, to achieve a meaningful outcome of benchmarking, it is imperative to have a realistic decision-making about the development endpoint of the NRA (whether it be a “gatekeeper” mitigating drug-related risks to the public health and/or as an “enabler” supporting research and innovation that weights on the different functionalities of the system).^10^ The complexity in operating benchmarking NRAs is further compounded by the different choices of reference points and tools for measuring functionalities, the means to collect data according to the predefined parameters, and the process of analysis in order to draw reliable comparison against the reference point. More importantly, it is the efforts put into learning and changes implementation based on evaluation results that matter in the quest for better capacities through benchmarking.

 Given the growing interests in benchmarking drug regulatory systems, it becomes highly relevant to see what research and country experiences has had to say about this phenomenon. Previous literature mainly focused on a number of aspects of regulatory benchmarking including: the introduction of the practice of benchmarking NRA^11^; the application of benchmarking NRAs for different purposes such as public health emergencies^12,13^ and pharmacovigilance^14,15^; various benchmarking tools or programmes such as the Global Benchmarking Tool (GBT) developed by the World Health Organization (WHO),^16^ and Optimizing Efficiencies in Regulatory Agencies (OpERA)^17^; and the country experiences of assessing and comparing regulatory capacities using benchmarking.^15,18-20^ However, such literature is yet to be systematically analyzed and reviewed to depict an overall research landscape about how regulatory capacities can be approved through benchmarking NRAs. This is especially concerning when considering that, according to the WHO, 70% of its member states are not able to effectively and efficiently regulate medical products in their nations, especially in many low- and middle-income countries.^21,22^

 As such, this study aimed to answer the following questions: What are the scopes of regulatory capacities covered by benchmarking NRAs? What tools are available for benchmarking regulatory capacities? What benefits have the NRAs seen from benchmarking regulatory capacities? And what is the most recent development in the benchmarking practices? For the purpose of this study, in consultation with the resolution WHA 67.20 by the WHO, benchmarking of regulatory systems implies “*a structured and documented process by which national drug regulatory authorities can identify and address gaps with the goal of reaching a level of regulatory oversight commensurate with a stable, well-functioning and integrated regulatory system.*”^23^

## Methods and Materials

 This study employed an integrative and critical review of the research on benchmarking drug regulatory capacities in compliance with the Preferred Reporting Items for Systematic Reviews and Meta-Analysis (PRISMA) guidelines.^24^ An integrative literature review was considered appropriate for the purpose of the study because it focused on combining, critiquing, and studying literature on the topic of benchmarking regulatory capacities in an integrated way in order to generate new frameworks and perspectives on this topic.^25^

###  Search Strategy

 Identification of the journal articles eligible for this review study consisted of the following steps. The primary concepts in this review included “benchmarking” and “regulatory capacity.” Based on preliminary research, the potential synonyms of “benchmarking” may include “benchmark,” “ranking,” “index,” “performance,” “indicator,” “evaluation,” and “assessment.” A pilot search for each term was conducted in PubMed and Web of Science to determine the frequency and relevance of each term. The four most frequent and relevant terms (“benchmark,” “ranking,” “index,” and “indicator”) were chosen to be included in the search strategy. Similarly, the terms “drug regulation,” “medicine regulation,” “regulatory authority,” “regulatory agency,” and “regulatory capacity” were used in the search strategy to reflect the concept of “regulatory capacity.” The search terms used in Chinese databases were: ((监管能力 OR 监管体系 OR 监管措施) AND (框架 OR 指标 OR 工具 OR 模型) AND (药品)).

 Six databases (PubMed, Scopus, Medline, Web of Science, Science Direct, and China National Knowledge Infrastructure) were searched for eligible literature since the database inception till 30 November 2022. To ensure an effective search, Medical Subject Headings terms, synonyms and keywords related to the two concepts were used to develop a comprehensive search strategy. Terms of each concept were combined using OR, then the two concepts were combined using AND. In addition, reference lists and citations of included literature were screened to identify possibly eligible studies for inclusion.

 Furthermore, international organizations and government agencies which had previously issued formal regulatory benchmarking tools or programmes were identified from the eligible studies and searched for eligible documents to be included in this study. The search covered the websites of such entities including the WHO (https://www.who.int/), the Organization for Economic Co-operation and Development (OECD) (https://www.oecd.org/), the Heads of Medicine Agencies (HMA) (https://www.hma.eu/), the Centre for Innovation in Regulatory Science (CIRS) (https://www.cirsci.org/), the US Government Accountability Office (GAO) (https://www.gao.gov/).

###  Inclusion and Exclusion Criteria

 Literature, published in either English or Chinese, was included if it directly discussed about benchmarking drug regulatory capacities, or employed benchmarking approach to compare different regulatory systems for the purpose of identifying gaps and making improvement. Literature which investigated, evaluated or compared drug regulatory systems without employing benchmarking approach or posing any direct or indirect implications for benchmarking practice were excluded.

###  Screening Process

 Literature was screened in compliance with the PRISMA statement. After removing the duplication, two authors (JS and XC) independently screened the titles and abstracts to identify literature that met the inclusion criteria. Full texts of potentially relevant articles were retrieved for detailed assessment. Discrepancies were discussed and resolved by agreement in consultation with 2 others authors (HH and COLU).

###  Data Extraction and Analysis

 The following data was extracted from the included articles into an Excel table: title, authors, year of publication, paper type/study design, purpose, underlying evaluation tools or frameworks, key indicators, current problems, major findings, and major implications/comments. For eligible studies, 2 authors (JS and XC) independently extracted data and any disagreements were resolved by seeking confirmation from another author (COLU). Different tools or programmes collected were verified and presented into seven criteria categories of the NRAs for capacity building, including: the name of tool/programme, issuing organization and time, scope of application, purpose, focus areas, compositions and quality assessment methods (if applicable). Such information was then used for in-depth and comparative analyses.

## Results

###  Literature Selection

**Figure 1:**
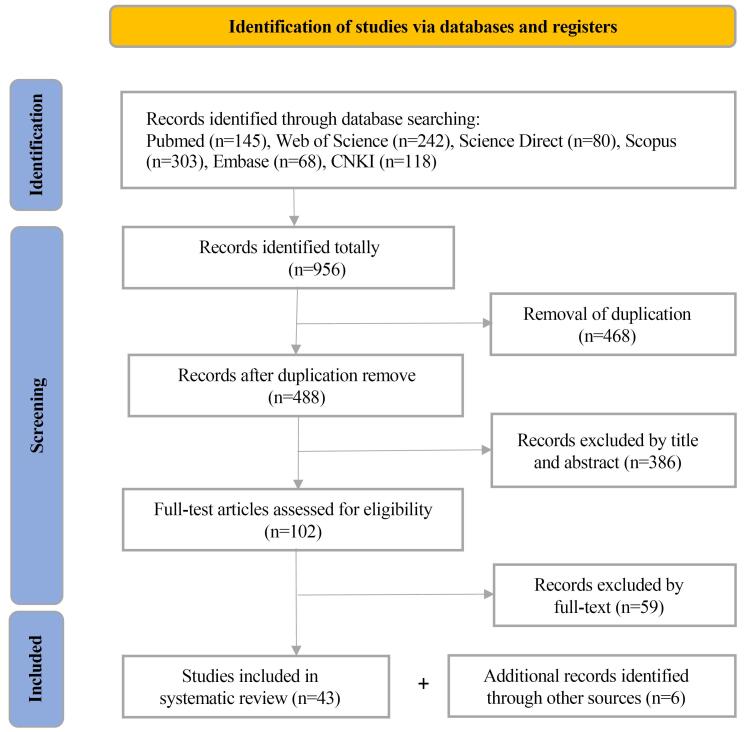
PRISMA Flowchart of Study Selection. Abbreviation: PRISMA, Preferred Reporting Items for Systematic Reviews and Meta-Analysis.

 Nine hundred and fifty-six records were identified from different databases, including 838 English initial records and 118 Chinese initial records. Upon removal of 468 duplicate articles, 488 records were proceeded to further screening by title and abstract, and, as a result, another 386 records were excluded. After full-text screening of the remaining 102 records, 59 records that did not related to benchmarking drug regulatory capacities were excluded. Ultimately, 43 eligible studies were included in this review (Figure 1). Furthermore, 6 documents about regulatory benchmarking tools or programmes retrieved from the websites of international organizations and government agencies were also included in this review for further analysis.

###  Literature Characteristics

 The 43 articles and 6 additional records included in this review were published between 2005 and 2022. The description of 43 articles is presented in Table 1.^11-15,18-20,26-60^ The research design included literature review (n = 3),^28,32,49^ expert interview (n = 1),^48^ empirical analysis (n = 2),^43,52^ comparative analysis (n = 7),^12,15,35,42,47,58,59^ retrospective analysis (n = 2),^19,41^ questionnaire (n = 10),^14,20,26,27,37-40,55,56^ description analysis (n = 6),^11,13,33,36,45,60^ and mixed methods (n = 12).^18,29-31,34,44,46,50,51,53,54,57^

**Table 1:** Description of Individual Studies Related to the Regulatory Capacity Building for National Regulatory Authorities

**Year, Authors**	**Country/Region**	**Study Design**	**Main Purpose**	**Underlying Tools or Frameworks**
2022, Bujar et al^26^	CIRS members	Questionnaire	Ensure quality, transparent, and consistent decision-making processes	Others: QoDoS
2022, Chaw et al^27^	Myanmar	Questionnaire	To assess the national regulatory system and regulatory activities with WHO-GBT indicators	GBT
2022, Garashi et al^28^	Developing countries	Literature review	To synthesise current research evaluating developing countries’ PV systems’ performance	Others: WHO-PV indicator
2022, Keyter et al^29^	South African	Comparative analysis, questionnaire	Develop a new regulatory review model for enhanced regulatory performance	GBT, OpERA, UMBRA
2022, Lavery et al^14^	Global	Structured benchmarking survey	To gain a better understanding of the impact of the pharmacovigilance system master file for MAHs	N/A
2022, Mahdavi et al^30^	Iran	Literature review and experts validating	To draw a roadmap for strengthening EIHP in Iran	Others: SAHSHA^a^ project: EIHP
2022, Owusu Sekyere et al^12^	Liberia, Sierra Leone, and the Gambia	Comparative analysis	Probed the outputs of capacity-strengthening activities for clinical trials oversight to take stock of progress made and examine remaining priorities	GBT; Others: GHPP RegTrain-VaccTrain
2022, Shabani et al^18^	Rwanda	Descriptive cross-sectional design with both quantitative and qualitative approaches	To assess the capacity of the Rwanda FDA	GBT
2022, Sithole et al^31^	Zimbabwe with Australia, Canada, Singapore, and Switzerland	Questionnaire, comparative analysis	To compare the medicines registration process of the Medicines Control Authority	N/A
2022, Xing et al^32^	China	Policy review	To improve the vaccine regulatory system in China	GBT
2022, Zhang et al^33^	China	Descriptive analysis	To introduce the third-party evaluation systems	GBT, OpERA
2021, Khadem Broojerdi et al^13^	WHO	Descriptive analysis	To analyse and document the current regulatory preparedness status, highlight the related gaps and challenges	GBT
2021, Li^34^	China	Dual organization theory, Delphi experts interview	To construct the drug emulation ability model	GBT, BEMA
2021, Rahalkar et al^20^	BRICS-TM with Australia, Canada, and Switzerland	Semi-quantitative questionnaire	To identify, compare, and evaluate regulatory requirements for the biosimilar development and review processes	N/A
2021, Rodier et al^35^	18 Maturing pharmaceutical markets	Comparative analysis	To determine current certificate of pharmaceutical product practices versus national regulatory guidelines	N/A
2021, Russom et al^36^	Eritrea	Descriptive analysis	To describe Eritrea’s success stories, key strategies for success, challenges encountered, and lessons learned	N/A
2021, Sithole et al^37^	Zimbabwe	Questionnaire	To assess the current regulatory review process of the Medicines Control Authority of Zimbabwe	OpERA
2020, Barry et al^15^	East Africa	Comparative assessment	To assess the functionality and identify the strengths and limitations of the national pharmacovigilance systems	The East African Community Harmonized Pharmacovigilance Indicators tool, GBT
2020, Guzman et al^11^	N/A	Descriptive analysis	To analyse the GBT key benefits for countries	GBT
2020, Hartmann et al^38^	Emerging countries	Questionnaire	To scale up global immunization, improve access to vaccines, and enhance scientific knowledge and operational efficiency in PV	ICH, EMA-GVP
2020, Keyter et al^39^	South Africa	Questionnaire	To identify criteria and current practices for implementing an abridged review process	Good reliance practices
2020, Liberti et al^40^	CARICOM region	Questionnaire	To understand the effectiveness and efficiency of the processes implemented by the Caribbean Regulatory System for the regulatory assessment of medicines for the region	OpERA
2020, Patel et al^41^	Brazil	Retrospective analysis	Analysis the timelines associated with important components of the ANVISA regulatory review process	OpERA
2020, Preston et al^42^	Small states	Assessment analysis	To strengthen the regulatory system	GBT
2020, Saaristo et al^43^	Finland	Empirical analysis	To analyse and test a theoretical generic health promotion capacity-building framework with empirical data on primary healthcare	Others: Health promotion capacity-building framework
2020, Sani et al^19^	Malaysia	Retrospective analysis	To provide NPRA with a breakdown of where the time is spent in their approval process	OpERA
2019, Keyter et al^44^	South African	Questionnaire, comparative analysis	To compare the registration process and the regulatory review model	N/A
2018, Chong et al^45^	APEC	Policy review	To identify appropriate regulatory practice and explores the feasible processes of regulatory convergence of APEC	N/A
2018, Mashaki Ceyhan et al^46^	Turkish	Questionnaire, comparative analysis	To assess the level of adherence to GRevP	N/A
2018, Tang et al^47^	China	Assessment analysis	To explore a method of construction of knowledge management system for drug evaluation and inspection	Knowledge management system
2017, Li et al^48^	Low- and middle-income countries	Stakeholder interview	Analysis the kinds of capacity needed to support decision makers when setting health priorities	Others: INNE Model
2017, Mery et al^49^	Canada	Systematic review	To identify key steps and elements considered for system-level evaluations of investment in quality improvement capacity building	N/A
2016, Liu et al^50^	China	Literature analysis, empirical studies	To establish regulatory capacity indicator system for social regulatory agencies to measure their regulatory capacity	Others: OECD 1995
2016, Zhang et al^51^	China	Literature survey, expert brainstorming, maximum difference scaling and internet questionnaire survey	To establish drug safety performance indicator system in Beijing	N/A
2015, Chen et al^52^	China	Empirical analysis	To establish an evaluation index system for the level of supervision of the circulation and safety of essential drugs in rural areas	N/A
2015, Yang et al^53^	China	Literature review, expert interview, content analysis and case study	To explore the definition, dimensions, and building mechanisms of drug regulatory capabilities and their relationship with regulatory performance	N/A
2014, Yao et al^54^	China	Literature and individual work experience	To explore the construction of drug regulatory core indicators in China	N/A
2014, Zhang et al^55^	China	Key stakeholder survey	To explore the model of evaluation on the ability of drug safety supervision in Beijing	Government performance theory
2013, Liu et al^56^	APEC member economies	Questionnaire	To assess the current use of GRevP	GRevP
2012, Yang et al^57^	China	Literature review, expert interview	To establish the indicator system for evaluating drug regulatory capacity in China	N/A
2009, McAuslane et al^58^	13 Countries in Asia, Latin America, the Middle East, and Africa	Comparative analysis	To record and analyse the regulatory procedures for the authorization of new medicines	N/A
2007, Hirako et al^59^	United States, Europe, Canada, Switzerland, and Australia	Comparative analysis	To identify and quantitate the stages of submission, review and regulatory action for NDA	N/A
2005, Cooke et al^60^	UK	Literature analysis	Measure the effectiveness of research capacity building in healthcare	Others: Research capacity building framework

Abbreviations: CIRS, Centre for Innovation in Regulatory Science; QoDoS, Quality of Decision-Making Orientation Scheme; GBT, Global Benchmarking Tool; WHO, World Health Organization; PV, Pharmacovigilance; UMBRA, Universal Methodology for Benefit-Risk Assessment; OpERA, Optimizing Efficiencies in Regulatory Agencies; EIHP, evidence-informed health policy-making; GHPP, Global Health Protection Programme; BEMA, Benchmarking of European Medicines Agencies; EMA, European Medicines Agency; CARICOM, Caribbean Community; ANVISA, Agência Nacional de Vigilância Sanitária; APEC, Asia-Pacific Economic Cooperation; GRevP, good review practices; OECD, Organization for Economic Co-operation and Development; FDA, Food and Drug Administration; BRICS-TM, Brazil, Russia, India, China, South Africa, Turkey, Mexico; MAHs, marketing authorization holders; N/A, not applicable; ICH, The International Council for Harmonisation of Technical Requirements for Pharmaceuticals for Human Use; GVP, Good Pharmacovigilance Practices; NPRA, National Pharmaceutical Regulatory Agency; NDA, New Drug Application. ^a^SASHA stands for evidence-informed health policy-making in Persian; INNE, Identification, Notification, and Evaluation of New Events.

 As reported in 22 of the 43 included studies, benchmarking had been employed to assess the drug regulatory system in a specific country including developed countries — including UK (n = 1), Canada (n = 1), and Finland (n = 1) — and developing countries — including China (n = 11), Eritrea (n = 1), Myanmar (n = 1), Rwanda (n = 1), Turkish (n = 1), Zimbabwe (n = 1), Malaysia (n = 1), Iran (n = 1), and Brazil (n = 1). Comparing drug regulatory systems using benchmarking at regional level had also been reported for the Caribbean Community (CARICOM) region (n = 1), East Africa (n = 1), South Africa (n = 3), and West Africa (n = 1). Furthermore, cross-country benchmarking based on international organizations, such as Asia-Pacific Economic Cooperation (APEC) (n = 2) and CIRS (n = 1), were also reported in 15 studies.

 In terms of benchmarking tools, 11 of the 43 included studies assessed the drug regulatory systems based on the GBT, of which 1 study combined indicators from the GBT and the Benchmarking of European Medicines Agencies (BEMA) (n = 1), 2 studies combined GBT indicators and OpERA tool (n = 2) and 2 studies combined GBT indicators and other indicators tool (n = 2). Four studies employed the OpERA tool to evaluated the NRAs’ regulatory capabilities and 14 studies employed other organizations evaluation indicators or methods. The remaining 16 studies used self-developed indicators or methods when conducting benchmarking.

###  The Main Themes of the Included Literature 

 After review and analysis of the included literature, 3 main themes related to benchmarking regulatory capabilities were identified: introduction of the concepts or methods of benchmarking; the application of benchmarking for internal assessment; and the application of benchmarking for external evaluation.

####  Introduction of the Concepts or Methods of Benchmarking

 Four studies introduced benchmarking and highlighted the advantages and benefits of applying benchmarking methods or tools in the assessment of regulatory capability. For instance, among these 4 studies, 2 of them focused on explaining the GBT,^15,27^ 1 study emphasised on the utilization of three-party evaluation systems,^33^ and 1 study explored the relationship between drug regulatory capabilities and regulatory performance.^53^

####  The application of Benchmarking for Internal Assessment 

 A total of 19 studies were conducted to internally assess the regulatory capabilities of NRAs. Of these, 6 studies were dedicated to observing the establishment of the national-level regulatory system.^18,30,35,50,54,57^ Additionally, 5 studies were designed to analyse the drug review process.^19,31,34,40,47^ Two studies focused on measuring the effectiveness of capability building in healthcare,^43,60^ while 3 studies concentrated on post-marketing surveillance,^51,52,55^ particularly regarding the circulation and safety of drugs. One study aimed to enhance the vaccine regulatory system in China,^32^ another aimed to evaluate the level of adherence to good review practices (GRevP),^46^ and 1 study aimed to identify the allocation of resources for capability building.^49^

####  The Application of Benchmarking for External Evaluation

 Drug regulatory capability of various countries or regions were investigated in 20 studies. At the national level, 4 studies aimed to evaluation the implementation of regulatory standards, including the Caribbean Regulatory System,^39^ GRevP,^56^ Certificate of pharmaceutical product,^42^ Pharmacovigilance (PV) system master file.^14^ Three studies aimed to evaluate the current regulatory situation evaluation and analyse the main challenge or problems faced by NRAs.^30,41,45^ Additionally, the capacity required for effective regulatory decision-making process was also a topic of interest in 2 studies.^26,48^ At the operation level, 7 studies were designed to identify the problems of the drug review process in the target area,^20,29,31,38,44,58,59^ of which four studies focused on the new drug registration process.^25,43,57,58^ The remaining 4 studies aimed to assess the regulatory functionalities related to pharmacovigilance,^28,37^ vaccines^11^ and clinical trials.^12^

###  Tools Used for Benchmarking

 According to the 6 additional records retrieved from WHO (n = 1),^16^ OECD (n = 1),^61^ HMA (n = 1),^62^ CIRS (n = 1),^17^ GAO (n = 2),^63,64^ a total of 5 benchmarking tools were identified (Table 2). These included the Global GBT Revision VI,^16^ the indicators of Regulatory Policy and Governance (iREG),^61^ the BEMA,^62^ OpERA programme,^17^ and the report evaluated the Workforce planning and Scientific-integrity-related procedures and training.^63,64^ The 5 tools or programs were issued from 2013 to 2022. The number of indicators included in these tools or programs range from 3 to 288.

**Table 2:** Description of Included Tools/Programmes Related to the Capacity Building

**Tool/Programme**	**Organization & Start Time**	**Scope of Application**	**Purpose**	**Focus Areas**	**Composition**	**Quality Assessment Methods**
GBT^16^	WHO, 2018	National regulatory systems	Identify strengths and areas for improvement; facilitate the formulation of an IDP to build upon strengths and address the identified gaps; prioritise IDP interventions; and monitor progress and achievements.	A variety of product types, including medicines, vaccines, blood products and medical devices.	9 Indicator categories, 9 regulatory functions, 268 (sub)indicators.	Maturity level, ranging from 1 to 4- no formal approach (level 1); reactive approach (level 2); stable, well-functioning system (level 3) and continual improvement emphasised (level 4).
iREG^61^	OECD, 2015	National policy areas in OECD member countries, not include practices at the sub-national level	Up-to-date evidence of OECD member countries’ regulatory policy and governance practices.	The processes of developing regulations that are carried out by the executive branch of the national Government.	3 Core areas, four sub-dimensions, 61 (sub)indicators.	Composite indicators are calculated as weighted averages of sub-indexes and vary between 0 and 6.
BEMA^62^	HMA, 2019	Systems and processes in individual agencies in EU/EEA	To contribute to the development of a world-class medicines regulatory system based on a network of agencies operating to best practice standards.	Management systems; Assessment of marketing authorisation applications; PV (drug safety) activities; and Inspection services.	12 Key performance indicator, 41 specific performance indicators.	Self-assessment and peer review assessment, and broadly based on ISO 9004 guidelines.
OpERA^17^	CIRS, 2013	National regulatory agencies	Help regulators integrate best practices that are fit-for-purpose for their remit, while ensuring the safety, efficacy and quality of their products.	Review performance goals and optimise review processes.	5 Performance metrics.	Country report and specific metrics collections, summary of review process timelines.
GAO analysis (Workforce planning & Scientific-integrity-related procedures and training)^63,64^	GAO, 2022	FDA	Help the government save money and work more efficiently and provide scientific advice on specific issues in the FDA's decision-making process.	Project-specific analysis.	3 Leading practice and 4 elements.	Data collection and analysis, Stakeholders interviews, etc.

Abbreviations: GBT, Global Benchmarking Tool; WHO, World Health Organization; IDP, institutional development plan; iREG, Indicators of Regulatory Policy and Governance; OECD, Organization for Economic Co-operation and Development; HMA, Heads of Medicine Agencies; BEMA, Benchmarking of European Medicines Agencies; EEA, European Economic Area; EU, European Union; PV, pharmacovigilance; OpERA, Optimising Efficiencies in Regulatory Agencies; CIRS, Centre for Innovation in Regulatory Science; GAO, Government Accountability Office; FDA, Food and Drug Administration.

 The benchmarking tools or programmes identified in this study were developed or recognised by a variety of international organizations, third-party professional organizations, and independent government departments, each with different purposes and focuses. Particularly, the GBT was developed to assess the national regulatory frameworks in terms of regulatory functions, while iREG was applicable to the investigation of the processes in relation to national regulatory policy. The tools issued by OpERA and GAO focused more specifically on the review processes and procedures. In addition, HMA was operated to liaise the regulatory frameworks of pharmaceutical product under the European Union (EU) and European Medicines Agency (EMA), while BEMA aimed to advance the standards of regulatory practices for individual member state bodies focusing on benchmarking management systems, drug authorization, pharmacovigilance, and inspection services. Further description of the assessment tools or programmes is presented in Table 2.

###  Functions, Indicators, and Sub-indicators Covered by the Benchmarking Tools 

 The GBT measured 9 functions across an overarching national regulatory system framework and regulatory functions (including national regulatory system; registration and marketing authorization; pharmacovigilance; market surveillance and control; licensing of establishments; regulatory inspections; laboratory testing; clinical trials oversight; and lot release of vaccines) by using 9 indicator categories (legal provisions, regulations and guidelines; organization and governance; policy and strategic planning; leadership and crisis management; quality and risk management system; resources; regulatory process; transparency, accountability and commination; and monitoring progress and assessing outcomes and impact) outlining 268 sub-indicators.

 The iREG indicators measured three key principles (including stakeholder engagement, regulatory impact analysis and ex post evaluation) using a total of 61 sub indicators. With BEMA, the key performance indicators and specific performance indicators were not separately listed but embedded in 14 sub-indicators of GBT and iREG. In the OpERA programme, the five indicators for evaluating the regulatory process of drug review and approval aligned with the GBT M06 (Mechanism in place to monitor regulatory performance and output), while the indicators for the workforce planning and scientific-integrity-related procedures and training were derived from the GBT RS06 (Human resources to perform regulatory activities) and RS10 (Mechanism in place to monitor regulatory performance and output).

**Figure 2:**
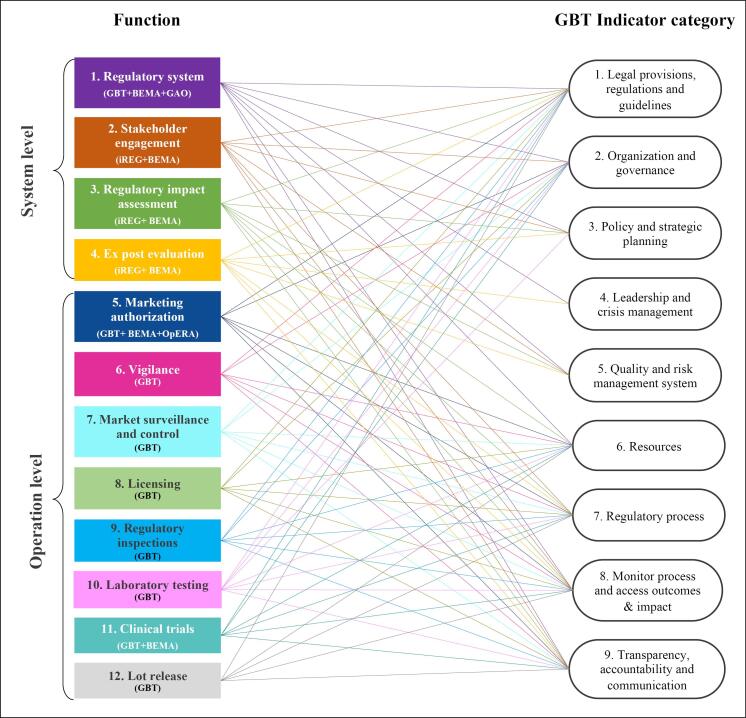
The Commonalities Across the Regulatory Benchmarking Tools and Programmes. The indicator categories are connected to the corresponding functions by lines of the same colors. Indicator categories with more connecting lines are those used more often to measure the regulatory functions. Abbreviations: GBT, Global Benchmarking Tool; iREG, Indicators of Regulatory Policy and Governance; BEMA, Benchmarking of European Medicines Agencies; OpERA, Optimising Efficiencies in Regulatory Agencies; GAO, Government Accountability Office.

 An integrative analysis of the above-mentioned 5 benchmarking tools and programmes provided an overall landscape of the benchmarking scope. As shown in Figure 2, an integrated benchmarking framework comprised of a total of 12 functions (including 4 at system level and 8 at operation level), 9 indicator categories and 382 sub-indicators.

 The commonalities across the regulatory benchmarking tools and programmes in terms of the functions and indicators covered are depicted in Figure 2. As shown, benchmarking “*1. Regulatory system*” at the system level and “*5. Marketing authorization*” at the operation level were of common interests to at least 3 benchmarking tools or programmes. Other functions such as “*2. Stakeholder engagement*,” “*3. Regulatory impact assessment,*” and “*4. Ex post evaluation*” at the system level and “*11. Clinical trials*” at the operation level were also of common interests to at least 2 benchmarking tools or programmes. Comparatively, the GBT was the most comprehensive benchmarking tool covering 9 out of the 12 functions.

 With regards to the indicators, it is also worth noting that all the indicators identified from the selected benchmarking tools or programmes corresponded to the 9 indicator categories in the GBT. This is demonstrated by the lines connecting the functions and the GBT indicator categories as shown in Figure 2. When considering the number of connecting lines of each of the GBT indicator category, it is also noted that the GBT indicator categories “*1. Legal provisions, regulations and guidelines*,” “*2. Organization and governance*,” “*6. Resources*,” “*7. Regulatory process*,” “*8. Monitor process and access outcomes *&* impact*,” and “*9. Transparency, accountability and communication*” were used most often to measure different functions of a regulatory system. More detailed information about the indicator categories used to measure the 12 functions of is provided in Table S1 of Supplementary file 1. Importantly, it can be seen that the assessment of any function in a drug regulatory system is a complex evaluation approach involving not one but multiple dimensions of indicators. More detailed description of the functions, indicators and sub-indicators is provided in Table S2.

###  Key Functions and Indicators Employed in the Benchmarking Studies 

 Among the 43 included studies, 15 studies covered multiple regulatory functions^11,13,15,18,20,27,32-34,42,44,45,50,54,57^ while the remaining 28 studies focused on only 1 function when evaluating the regulatory capacities of NRAs.^12,14,19,26,28-31,35-41,43,46-49,51-53,55,56,58-60^ Apart from 6 of the studies^11,13,18,27,32,42^ which fully adopted the 9 GBT indicator categories when evaluating the functions of interest, the remaining studies adopted only some of the GBT indicator categories and sub-indicators whenever deemed relevant by the researchers. In 5 studies,^12,15,29,33,34^ a combination of indicators from different benchmarking tools or programmes were used as measurements of the regulatory capacities. More detailed description about the key functions and indicators employed in the benchmarking studies is provided Table S3.

###  Most Common Problems and Recommended Actions Based on Benchmarking Results

 Among the 43 studies, some common problems or challenges in drug regulation had been repeatedly reported and, in some occasions, recommended actions based on the benchmarking results had been proposed accordingly. As shown in Table 3, there were 6 key aspects identified as the major areas of concern which included: legal provision; regulatory process; resources; cooperation and communication; and stakeholder engagement.

**Table 3:** Most Common Regulatory Problems and Recommended Actions Identified From Benchmarking Results

**Key Areas of Benchmarking Regulatory Capacity**	**Common Regulatory Problems**	**Specific Recommended Actions to the Problem**
Legal prevision	Inflexible regulatory policies or guidelines (n = 2)^50,51^	Policy and legal framework (n = 1)^40^
	Lack of pharmacovigilance system (n = 2)^28,36^	
	Lack of framework of emergency preparedness (n = 1)^12^	Application strategies/guideline/framework (n = 10)^12,13,27,29,31,32,38,50,53,56^
	Lack of the quality management system (n = 1)^18^	
Regulatory process	Review time exceed the agency’s overall target time or the international average time (n = 6)^19,31,40,41,44,46^	Review time milestone(n = 8)^19,29,31,35,37,41,44,46^
	Inefficient drug safety supervision (n = 4)^51,52,54,55^	
	Need for improvement of decision-making practices (n = 2)^26,37^	Evidence-based decision-making practices (n = 5)^26,29,30,31,37^
	Lack of quality measure or risk-based evaluation (n = 2)^28,29^	Regulatory process and practice (n = 22)^15,18-20,29,31-33,35,37,39-41,44,46,47,51,52,56-59^
	Insufficient independence of regulators (n = 1)^50^	
	Lack of centralised functions and powers (n = 1)^50^	
	Single regulatory tools (n = 1)^50^	
	Unclear level of performance appraisals (n = 1)^52^	Performance appraisal (n = 7)^32,38,45,52,53,55,57^
	Challenge of setting the priority areas (n = 1)^48^	Fast-track/accelerated reviews (n = 1)^42^
	Informal implementation of GRevP (n = 2)^46,56^	Good review practice (n = 2)^44,46^
Resources	Lack of training and education (n = 7)^18,29,39,47,48,53,57^	Research and training (n = 7)^18,27,38,48,49,56,60^
	Insufficient human resources (n = 5)^18,27,39,40,42^	Human resources staffing (n = 5)^18,28,30,48,60^
	Insufficient financial resources (n = 2)^42,49^	
	Lack of regulatory inspection tools/equipments (n = 1)^18^	Equipment and tools, automation systems (n = 5)^18,28,30,52,60^
	Lack of enough capacity of the quality control laboratory (n = 1)^18^	
	Insufficient innovation technologies (n = 2)^18,29^	Digitization (online submission/database/Develop algorithms) (n = 6)^28,20,31,38,42,52^
Cooperation and communication	Lagging transparency and communication (n = 3)^14,29,31^	Collaboration/networks (n = 19)^12,14,26,28,29,31,32,35,38,39,42-46,48,52,56,60^
	Absence of reliance approach and participation in harmonization activities (n = 2)^20,38^	
Stakeholder engagement	Limited stakeholder involvement and engagement (n = 1)^37^	Relevant stakeholders’ participation (n = 5)^14,15,28,38,48^
Others	Gaps between the academic outcomes of publications in peer reviewed journals or successful grant applications and the resolution of the regulatory practices (n = 1)^60^	

Abbreviation: GRevP, good review practices.

 With respect to legal prevision, 6 studies identified four main types of regulatory issues including the lack of flexible regulatory policies or guidelines (n = 2),^50,51^ pharmacovigilance systems (n = 2),^28,36^ the framework of emergence preparedness (n = 1),^12^ and a lack of quality management systems (n = 1).^18^ Correspondingly, 1 study recommended advancing the establishment of policy and legal framework,^40^ while 10 studies focused on the promotion of practical strategies and guideline.^12,13,27,29,31,32,38,50,53,56^

 Regarding the regulatory processes, 17 studies identified 10 common problems, with a significant emphasis on the prolonged product review time (n = 6),^19,31,40,41,44,46^ and 8 studies also pointed to setting milestone for review time.^19,29,31,35,37,41,44,46^ Twenty-two studies sought to optimise the regulatory processes and practice,^5,18-20,29,31-33,35,37,39-41,44,46,47,51,52,56-59^ but did not provide specific implementation details.

 The shortage of human resources,^18,27,39,40,42^ training or education^18,29,39,47,48,53,57^ financial resources^42,49^ and equipment or technique resources^18,29^ were reported to exert a negative impact on regulation. In addition, the lack of transparency and communication,^14,29,31^ as well as the lack of involvement of key stakeholders,^37^ were identified as common problems upon benchmarking. Nineteen studies proposed enhancing communication and cooperation at all levels^12,14,26,28,29,31,32,35,38,39,42-46,48,52,56,60^ while 5 studies encouraged stakeholder participation to drive regulatory decision-making.^14,15,28,38,48^

 Moreover, 1 study mentioned the gaps between the academic outcomes of publications in peer reviewed journals or successful grant applications and the resolution of regulatory practices.^60^ Nevertheless, it is worth noting that not every common problem was addressed with specific recommended action.

###  Anticipated Outcomes of Benchmarking Reported in the Included Studies

 Eighteen studies assessed the outcomes when applying benchmarking for regulatory improvement, including the promotion of regulatory reliance and harmonization (n = 7),^11,13,14,28,35,39,45^ the enhancement of regulatory transparency (n = 4),^27,29,31,44^ the reducing of timelines and improving patients’ access to new medicines (n = 3),^11,29,32^ the optimization of publicly available information (n = 3),^27,44,52^ and the improvement of pharmaceutical trade (n = 1).^11^

## Discussion

**Figure 3:**
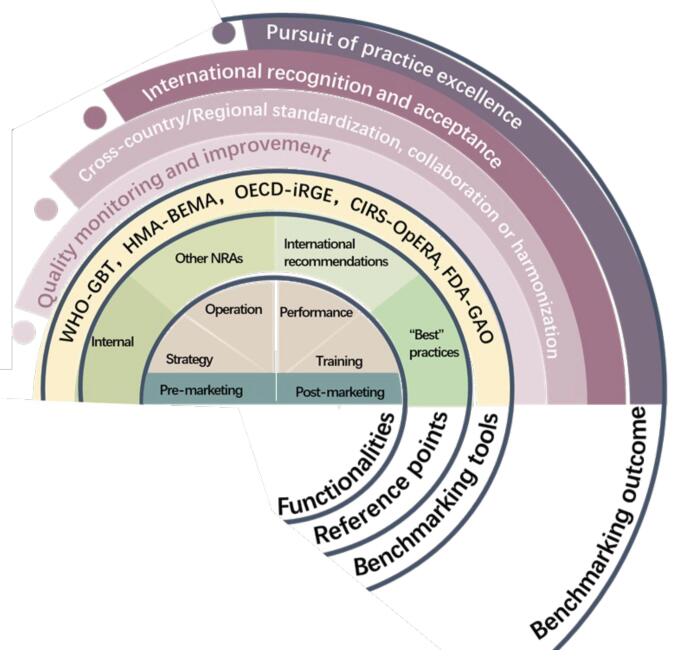
Decision-Making Framework for Benchmarking Drug Regulatory Capacities. Abbreviations: WHO, World Health Organization; GBT, Global Benchmarking Tool; HMA, Heads of Medicine Agencies; BEMA, Benchmarking of European Medicines Agencies; OECD, Organization for Economic Co-operation and Development; iREG, Indicators of Regulatory Policy and Governance; CIRS, Centre for Innovation in Regulatory Science; OpERA, Optimizing Efficiencies in Regulatory Agencies; FDA, Food and Drug Administration; GAO, Government Accountability Office.

 This literature review reaffirms that benchmarking has been employed by many NRAs as an important strategy in quality monitoring and management in pursuit of improvement in regulatory capacities. Further analysis of the included literature has depicted an overall research landscape on this phenomenon covering the main purposes of benchmarking, different benchmarking tools and comparison of the corresponding indicators, key indicators selected for benchmarking, major areas of improvement based on benchmarking results, most common recommended actions following up benchmarking practices, and anticipated of benchmarking outcomes. An integrative analysis of such findings gave rise to a framework for decision-makers in NRAs when deciding why and how benchmarking should be undertaken (Figure 3) which will be further discussed in the following. Nevertheless, the literature included in this review did not provide any empirical findings showing that NRAs had benefited from benchmarking regulatory capacities.

###  The Decision-Making Framework of Benchmarking 

 Using benchmarking to guide the advancement of pharmaceutical regulatory system echoed with the increasing emphasis of policy-makers on “evidence-informed health policy-making” (EIHP) to inform the decision-making in the contemporary healthcare.^65,66^ The EIHP approach aims to fully inform the best available research evidence as an input to the healthcare policy-making process. As shown in the current review, more and more NRAs currently include the benchmarking results as one of evidence in their regulatory capacity monitoring and management.

 As shown in Figure 3, the decision-making about benchmarking regulatory capacities involved multifaceted considerations of the benchmarking scope in terms of functionalities, the choice of benchmarking tools and reference points which would collectively determine the benchmarking outcome. At the functionality level, the benchmarking scope may encompass “strategy” (focus on the strategic goals and leadership of advancing NRAs such as the vaccine regulatory system^32^), “operation” (focus on the key processes to eliminate the weaknesses of regulation such as the PV system,^14,28,36,38^ “performance” (focus on the key performance indicators such as the delays in assessing applications owing to the staff manpower^40^) and “training” (focus on priority areas of capacity building such as workforce planning and training^17,29^). Another perspective when deciding on the scope might refer to pre-marketing and post-marketing functionalities of an NRA.

 The next important consideration when conducting benchmarking in drug regulation practice is the choice of reference points. When benchmarking was conducted internally, previous benchmarking results could be used as a baseline for continuous monitoring of performance to identify any changes in regulatory practice over time. Cross-country benchmarking, on the other hand, could be used to inform actions for regulatory practice standardization that promotes collaboration and harmonization across a region or a consortium. Coordinated efforts across NRAs in improving regulatory practice in common areas have been recognised as an important measure to facilitate regulatory reliance and harmonization at regional level.^11,13,39,45^

 It was also found that benchmarking was conducted with different tools and indicators which were selected based on the functionalities of interests. A range of benchmarking tools have been made available and it remained at decision-maker’s discretion about the choice of tools and the combination of indicators from readily available benchmarking tools or self-developed initiative as they were deemed fit the purpose of benchmarking.

 Comprehensive benchmarking against an international benchmarking framework would further benefit the credibility and international recognition of the NRAs. For instance, following Singapore which reached Maturity Level 4 in medicines in February 2022 which is the highest level achievable for regulatory system evaluation against the WHO’s GBT, the Ministry of Food and Drug Safety of the Republic of Korea also announced in November 2022 that it had reached Maturity Level 4, in both medicines and vaccines regulations.^67^ Countries with Maturity Level 3 or Maturity Level 4 according to the GBT are eligible to become a WHO listed authority so that they may be considered as a reference point by other regulatory authorities for reaching own decisions in approving medical products.^68^ For NRAs to be evaluated and recognised as operating at an advanced level of performance with continuous improvement is pertained to profound both practical and signifying implications at an international level.^67^ Benchmarking against best practices would further help NRAs to achieve regulatory excellence.

###  Benchmarking as a Process From Bench-Learning to Bench-Action

 It is worth noting that while benchmarking is important in identifying gaps and weakness, it is the “bench-learning” and “bench-action” that are key to making changes.^69,70^ However, little has been reported about communicating the aftermaths of benchmarking exercises and how to divert the findings and knowledge between and among researchers and NRAs to advance performance and address the gaps. All the included studies rested on the monologues about the relevance of benchmarking.

 Decisions about employing benchmarking requires systematic planning and multifaceted perspectives (including but not limited to the political environment, the latest advances in pharmaceutical research and development, the unmet needs of the patients, the availability of high-level engagement and resources, etc) to formulate effective implementation approaches. Addressing the gaps in regulatory performance is part of a highly complex undertaking involving not just NRAs but also other counterparts in the pharmaceutical system, as well as other counterparts in the larger environment of health system, both locally and beyond.^71^ However, it appeared in this review that benchmarking often took place in silos with no significant engagement of researchers or other government agencies with the NRAs on following up the findings and solving critical gaps.

 Indeed, as revealed in this review, barriers to implementing changes might be multifaceted encompassing, just to name a few, the political environment, the deficit in the information systems, the scarcity of related research to form the scientific foundation, and the lack of continuous engagement of leadership. Decisions about interventions that bring changes to drug regulatory practices often warrants the guidance from high-level governance to identify effective approaches and thus systematic planning.

 To move this facet of practice forward, a systems thinking approach guided by implementation science might offer a roadmap that help translate the benchmarking findings into formulation of actions for facilitating changes. Systems thinking is an approach that advocates for the involvement of key stakeholders to map the drug regulatory system, identify where the key impediments lie, and design synergistic and system-ready pathway towards benchmarking practice.^72^ This approach calls for transdisciplinary and translational approaches and encourages relationship-building across various functionalities of the NRAs so as to achieve a common set of relevant goals and objectives on drug regulation.

 Identifying the strengths and weaknesses in drug regulatory capacities through benchmarking using “systems thinking” approach then leads to the need for a coordinated and collaborative effort to implement and sustain changes in regulatory measure. Nevertheless, major challenges for bench-actions to be translated into sustained routine practice are foreseeable. There needs to be a scientific approach to identify the range of factors that are likely to facilitate the uptake of recommended actions and changes in regulatory practice, and to plan and act accordingly. More importantly, regulatory management systems to measure changes and demonstrate any outcomes associated with changes in regulatory capacities related to benchmarking is essential to support the sustainable development of the intervention or service. For this, implementation science knowledge and strategies must be employed and incorporate into the regulatory management systems to promote intervention validity, while collecting the data necessary for establishing evidence-based improvement to bargain for continuous resources input for benchmarking exercises.^73^

###  Limitations of the Study Findings 

 This study provides a comprehensive view of benchmarking the NRAs for capacity building in term of the existing tools, practices, and recommendations based on literature retrieval, analysis and data synthesis. Nevertheless, our review has some limitations. The first limitation is that we were not able to define a uniform system to determine the “maturity level” for all indicators due to the heterogenicity of the assessment methods employed in each benchmarking tool or programme included in this study. For each indicator of regulatory capacity, the maturity level is important not only for reflecting the status but also for measuring progress. Drawing on the successful experiences of the work on the GBT by the WHO, future research is warranted to yield specific criteria of quality assessment methods for each indicator. Another limitation of this review may be contributed by publication bias. The risks of negative outcomes about benchmarking regulatory capacity being rarely or unlikely to be fully reported in the literature cannot be ruled out possibly compromising the comprehensiveness of the overall research landscape about regulatory benchmarking presented in this review. Furthermore, considering that not all benchmarking tools or programs, and the related regulatory performance data are publicly available, the limitation in full access to all regulatory benchmarking information may inevitably affect the completeness of the findings reported in this review.

## Conclusion

 Benchmarking drug regulatory capacities is a complex process that has been increasingly adopted by NRAs for measuring the regulatory performance and monitoring the progress. This review has analysed in detail the “why” and “how” to employ benchmarking to improve regulatory practice. For effective benchmarking that leads to bench-learning and bench-action, well-informed decisions about the goals, the scope, the choice of reference points and benchmarking tools are essential to guide the implementation strategies, coordination of resources, and stakeholders’ participation and cooperation. Nevertheless, the evidence for the possible benefits of benchmarking remains scarce. There is a need for more empirical studies to develop evidence about how benchmarking can improve drug regulatory capacities.

## Ethical issues

 Not applicable.

## Competing interests

 Authors declare that they have no competing interests.

## Funding

 This work was supported by a grant from University of Macau (reference numbers: SRG2021-00007- ICMS and MYRG2022-00229-ICMS).

## Supplementary files

Supplementary file 1 contains Tables S1-S3.
