# Supplementary material for: Benchmarking Drug Regulatory Systems for Capacity Building: An Integrative Review of Tools, Practice, and Recommendations
Source: Int J Health Policy Manag. 2023 Oct 16;12:8100. doi: 10.34172/ijhpm.2023.8100 (PMC10699822; doi:10.34172/ijhpm.2023.8100)
Supplement: Supplementary file 1 — contains Tables S1-S3. [file ijhpm-12-8100-s001.pdf]

Article title: Benchmarking Drug Regulatory Systems for Capacity Building: An Integrative Review of Tools, Practice, and Recommendations

Journal name: International Journal of Health Policy and Management (IJHPM)

Authors' information: Junnan Shi[1]¶, Xianwen Chen[1]¶, Hao Hu[1,2,3], Carolina Oi Lam Ung[1,2,3]*

[1]State Key Laboratory of Quality Research in Chinese Medicine, Institute of Chinese Medical Sciences, University of Macau, Macao, China.

[2]Centre for Pharmaceutical Regulatory Sciences, University of Macau, Macao, China.

[3]Department of Public Health and Medicinal Administration, Faculty of Health Sciences, University of Macau, Macao, China.

***Correspondence to:** Carolina Oi Lam Ung; Email: carolinaung@um.edu.mo

Citation: Shi J, Chen X, Hu H, Ung COL. Benchmarking drug regulatory systems for capacity building: an integrative review of tools, practice, and recommendations**.** Int J Health Policy Manag. 2023;12:8100. doi:10.34172/ijhpm.2023.8100

Supplementary file 1

Table S1. The indicator system of included benchmarking tools

Category / Function	01 Legal provisions, regulations and guidelines	02 Organization and governance	03 Policy and strategic planing	04 Leadership and crisis management	05 Quality and risk management system	06 Resources	07 Regulatory process	08 Monitor process and access outcomes & impact	09 Transparency, accountability and communication
RS	✓	✓	✓	✓	✓	✓		✓	✓
MA	✓	✓				✓	✓	✓	✓
VL	✓	✓				✓	✓	✓	✓
MC	✓	✓				✓	✓	✓	✓
LI	✓	✓				✓	✓	✓	✓
RI	✓	✓				✓	✓	✓	✓
LT	✓	✓	✓			✓	✓	✓	✓
CT	✓	✓				✓	✓	✓	✓
LR	✓	✓				✓	✓	✓	✓
SE	✓	✓	✓		✓		✓	✓	✓
RIA	✓		✓		✓	✓	✓	✓	✓
ePE	✓		✓		✓	✓	✓	✓	✓

Abbreviations: RS, National Regulatory System; SE, Stakeholder engagement; RIA: Regulatory Impact Assessment; ePE: ex Post Evaluation; MA, Registration and Marketing Authorization (MA); VL, Vigilance; MC, Market Surveillance and Control; LI, Licensing Establishments; RI, Regulatory Inspection; LT: Laboratory Testing; CT: Clinical Trials Oversight; LR: NRA Lot Release.

Table S2. Comparison of the capacity indicators of different tools/programmes

Function	Category	Indicator	No. sub-indicator	WHO	BEMA	iREG	OpERA	GAO
01 National regulatory system (RS)	Legal provisions, regulations and guidelines	RS01 Legal provisions, regulations and guidelines required to define regulatory framework of national regulatory system.	9	●				
	Organization and governance	RS02 Arrangement for effective organization and good governance.	4	●				
	Policy and strategic planning	RS03 Strategic plan with clarified objective in place.	5	●	◎			
	Leadership and crisis management	RS04 Regulatory system is supported with leadership and crisis management plans.	5+3*	●	◎			
	Quality and risk management system	RS05 Quality management systems (QMS) including the risk management principles are applied and realized.	14	●	◎			
	Resources (HR, FR, infrastructure and equipment)	RS06 Human resources to perform regulatory activities.	4+1*	●	◎			■
		RS07 Financial resources to perform regulatory activities.	5	●	◎			
		RS08 Infrastructure and equipment to perform regulatory activities.	3+2*	●	◎			
	Transparency, accountability and communication	RS09 Mechanisms exist to promote transparency, accountability and communication.	9+3*	●	◎			
	Monitoring progress and assessing outcomes & impact	RS10 Mechanism in place to monitor regulatory performance and output.	2+5*	●	◎			■
02 Registration and Marketing Authorization (MA)	Legal provisions, regulations and guidelines	MA01 Legal provisions, regulations and guidelines required to define regulatory framework of registration and/or marketing authorization.	13	●				
	Organization and governance	MA02 Arrangement for effective organization and good governance.	2	●				
	Resources (HR, FR, infrastructure and equipment)	MA03 Human resources to perform registration and marketing authorization activities.	4	●				
	Regulatory process	MA04 Procedures established and implemented to perform registration and/or marketing authorization.	10	●				
	Transparency, accountability and communication	MA05 Mechanism exists to promote transparency, accountability and communication.	4	●				
	Monitoring progress and assessing outcomes & impact	MA06 Mechanism in place to monitor regulatory performance and output.	2	●	◎		◆	
03 Vigilance (VL)	Legal provisions, regulations and guidelines	VL01 Legal provisions, regulations and guidelines required to define regulatory framework of vigilance.	7	●				
	Organization and governance	VL02 Arrangement for effective organization and good governance.	2	●				

	Resources (HR, FR, infrastructure and equipment)	VL03 Human resources to perform vigilance activities.	4	●
	Regulatory process	VL04 Procedures established and implemented to perform vigilance activities.	8	●
	Monitoring progress and assessing outcomes & impact	VL05 Mechanism in place to monitor regulatory performance and output.	2	●
	Transparency, accountability and communication	VL06 Mechanism exists to promote transparency, accountability and communication.	3	●
04 Market Surveillance and Control (MC)	Legal provisions, regulations and guidelines	MC01 Legal provisions, regulations and guidelines required to define regulatory framework of market surveillance and control activities.	7	●
	Organization and governance	MC02 Arrangement for effective organization and good governance.	2	●
	Resources (HR, FR, infrastructure and equipment)	MC03 Human resources to perform market surveillance and control activities.	4	●
	Regulatory process	MC04 Procedures established and implemented to perform market surveillance and control	8	●
	Monitoring progress and assessing outcomes & impact	MC05 Mechanism in place to monitor regulatory performance and output.	3	●
	Transparency, accountability and communication	MC06 Mechanism exists to promote transparency, accountability and communication.	3	●
05 Licensing Establishments (LI)	Legal provisions, regulations and guidelines	LI01 Legal provisions, regulations and guidelines required to define framework for licensing activities.	5	●
	Organization and governance	LI02 Arrangement for effective organization and good governance.	2	●
	Resources (HR, FR, infrastructure and equipment)	LI03 Human resources to perform licensing activities.	4	●
	Regulatory process	LI04 Procedures established and implemented to perform licensing activities.	4	●
	Monitoring progress and assessing outcomes and impact	LI05 Mechanism in place to monitor regulatory performance and output.	2	●
	Transparency, accountability and communication	LI06 Mechanism exists to promote transparency, accountability and communication.	2	●
06 Regulatory Inspection (RI)	Legal provisions, regulations and guidelines	RI01 Legal provisions, regulations and guidelines required to define regulatory framework of inspection and enforcement.	5	●
	Organization and good governance	RI02 Arrangement for effective organization and good governance.	2	●
	Resources (HR, FR, infrastructure and equipment)	RI03 Human resources to perform regulatory inspection activities.	4	●

	Regulatory process	RI04 Procedures established and implemented to perform inspection and enforcement.	6	●	
	Monitoring progress and assessing outcomes & impact	RI05 Mechanism in place to monitor regulatory performance and output.	5	●	
	Transparency, accountability and communication	RI06 Mechanism exists to promote transparency, accountability and communication.	4	●	
07 Laboratory Testing (LT)	Legal provisions, regulations and guidelines	LT01 Legal provisions, regulations and guidelines required to define the regulatory framework of laboratory testing activities.	2	●	
	Organization and governance	LT02 Arrangement for effective organization and good governance.	2	●	
	Policy and strategic planning	LT03 Laboratory activities implemented as per well-established plans and policies according a Quality Management System (QMS).	4	●	
	Resources (HR, FR, infrastructure and equipment)	LT04 Human resources to perform laboratory testing activities.	4	●	
		LT05 Well maintained and equipped infrastructures for laboratory activities.	2	●	
	Regulatory process	LT06 Procedures established and implemented to perform laboratory testing activities according to Quality Management System.	5	●	
	Transparency, accountability and communication	LT07 Mechanism exists to promote transparency, accountability and communication.	1	●	
	Monitoring progress and assessing outcomes & impact	LT08 Mechanism in place to monitor regulatory performance and output.	4	●	
	Policy and strategic planning	LT09 Measures for occupational health and safety.	3	●	
	Regulatory process	LT10 Measures for good management of outsourced laboratory activities.	1	●	
08 Clinical Trials Oversight (CT)	Legal provisions, regulations and guidelines	CT01 Legal provisions, regulations and guidelines required to define regulatory framework of clinical trials oversight.	11	●	
	Organization and governance	CT02 Arrangement for effective organization and good governance.	2	●	
	Resources (HR, FR, infrastructure and equipment)	CT03 Human resources to perform clinical trials oversight activities.	4	●	
	Regulatory process	CT04 Procedures established and implemented to perform clinical trials oversight.	7	●	
	Transparency, accountability and communication	CT05 Mechanism exists to promote transparency, accountability and communication.	2	●	
	Monitoring progress and assessing outcomes and impact	CT06 Mechanism in place to monitor regulatory performance and output.	4	●	◎

09 NRA Lot Release (LR)	Legal provisions, regulations and guidelines	LR01 Legal provisions, regulations and guidelines required to define regulatory framework of independent lot release by the NRA.	2	●		
	Legal provisions, regulations and guidelines	LR02 Arrangement for effective organization and good governance.	2	●		
	Resources (HR, FR, infrastructure and equipment)	LR03 Human resources to perform NRA lot release.	4	●		
	Regulatory process	LR04 Procedures established and implemented to perform NRA lot release.	3	●		
	Transparency, accountability and communication	LR05 Mechanism for information-sharing exists to promote transparency and accountability.	2	●		
	Monitoring progress and assessing outcomes and impact	LR06 Mechanism in place to monitor regulatory performance and output.	4	●		
10 Stakeholder engagement (SE)	Methodology	SE01 Consultation open to the general public: during early stages of developing regulations	NA			▲
		SE02 Consultation open to the general public: during later stages of developing regulations	NA			▲
		SE03 Guidance	NA			▲
		SE04 Methods of stakeholder engagement adopted in early stages of developing regulations	NA			▲
		SE05 Methods of stakeholder engagement adopted in later-stages of developing regulations	NA			▲
		SE06 Minimum periods	NA			▲
		SE07 Use of interactive websites	NA			▲
		SE08 Formal requirements	NA			▲
	Systematic adoption	SE09 Stakeholder engagement conducted in practice in early stages of developing regulations	NA		◎	▲
		SE10 Stakeholder engagement conducted in practice in later stages of developing regulations	NA		◎	▲
	Oversight and Quality Control	SE11 Oversight and quality control function	NA			▲
		SE12 Publically available evaluation of stakeholder engagement	NA			▲
	Transparency	SE13 Transparency of process	NA			▲
		SE14 Consultations are made open to general public	NA			▲
		SE15 Consideration and response to stakeholder comments	NA			▲
		SE16 Availability of information	NA			▲
	Methodology	RIA01 Assessment of budget and public sector impacts	NA			▲
		RIA02 Assessment of competition impacts	NA			▲

		RIA03 Assessment of other economic impacts	NA		▲
		RIA04 Assessment of other impacts	NA		▲
		RIA05 Assessment of environmental impacts	NA		▲
		RIA06 Assessment of social impacts	NA		▲
		RIA07 Assessment of distributional effects	NA		▲
		RIA08 Assessment of wider cost (e.g. macroeconomic costs)	NA		▲
		RIA09 Benefits identified for specific groups	NA		▲
		RIA10 Consideration of issues of compliance and enforcement	NA		▲
		RIA11 Costs identified for specific groups	NA		▲
		RIA12 Guidance	NA		▲
		RIA13 Identify and assess regulatory options	NA		▲
11 Regulatory Impact Assessment (RIA)		RIA14 Requirement to identify benefits	NA		▲
		RIA15 Requirement to identify costs	NA		▲
		RIA16 Requirement to identify process of assessing progress in achieving regulation's goals	NA		▲
		RIA17 Requirement to qualitatively assess benefits	NA		▲
		RIA18 Requirement to quantify benefits	NA		▲
		RIA19 Requirement to quantify costs	NA		▲
		RIA20 Risk assessment	NA		▲
		RIA21 Types of costs quantified	NA		▲
		RIA22 Formal requirements	NA		▲
	Systematic adoption	RIA23 RIA conducted in practice	NA		▲
		RIA24 Proportionality	NA		▲
		RIA25 Oversight	NA		▲
	Oversight and Quality Control	RIA26 Publically available evaluation of RIA	NA		▲
		RIA27 Quality control	NA	◎	▲
	Transparency	RIA28 Responsibility and transparency	NA		▲
		RIA29 Transparency of Process	NA		▲

12 ex Post Evaluation (ePE)		ePE01 Assessment of costs and benefits	NA		▲
		ePE02 Assessment of achievement of goals	NA		▲
	Methodology	ePE03 Assessment of impacts	NA		▲
		ePE04 Assessment of consistency with other regulations	NA		▲
		ePE05 Established methodologies and guidance	NA		▲
		ePE06 Use of mechanisms for review including ad hoc reviews	NA		▲
		ePE07 Formal requirements	NA		▲
	Systematic adoption	ePE08 ex post evaluations conducted in practice	NA		▲
		ePE09 In-depth reviews	NA		▲
		ePE10 Presence of standing body	NA		▲
		ePE11 Proportionality	NA		▲
	Oversight and Quality Control	ePE12 Oversight and quality control function	NA	◎	▲
		ePE13 Publically available evaluation of ex post evaluation	NA		▲
		ePE14 Ongoing stakeholder engagement	NA		▲
	Transparency	ePE15 Stakeholder engagement	NA		▲
		ePE16 Transparency of process	NA		▲

*: sub indicators that can integrated with the indicators from other assessment tools or programmes; RS: National regulatory system; MA: Registration and Marketing Authorization; VL: Vigilance; MC: Market Surveillance and Control; LI: Licensing Establishments; RI: Regulatory Inspection; LT: Laboratory Testing; CT : Clinical Trials Oversight; LR: NRA Lot Release; SE: Stakeholder engagement; RIA: Regulatory Impact Assessment; ePE: ex Post Evaluation; ●: cover indicators of WHO-GBT; ◎: cover indicators of HMA-BEMA; ▲: cover indicators of OECD iREG; ◆: cover indicators of CIRS OpERA; ■: cover indicators of FDA GAO.

Table S3. Key functions and indicators identified from the included 43 research articles that evaluated regulatory capacities using benchmarking approach

No.	Year, authors	Function/s under investigation	Key indicators used to measure regulatory capacities
**1**	2022, Chaw et al. [27]	Multiple	RS, MA, VL, MC, LI, RI, LT, CT, LR
**2**	2022, Shabani, J.B.B., et al.[18]	Multiple	RS, MA, VL, MC, LI, RI, LT, CT, LR
**3**	2022, Xing LY, et al. [32]	Multiple	RS, MA, VL, MC, LI, RI, LT, CT, LR
**4**	2022, Zhang Q, et al. [33]	Multiple	RS, MA, VL, MC, LI, RI, LT, CT, LR
**5**	2021, Khadem Broojerdi A, et al. [13]	Multiple	RS 03.04, RS 04.05, RS 07.03, MA 01.06, MA01.08, MA 01.12, MA 04.07,VL 04.06,RI 01.05,CT 01.11
**6**	2021, Li F. [34]	Multiple	RS 03-10; MA 01-06; CT 06; SE 09-10; RIA 27; ePE 12
**7**	2021, Rahalkar H, et al. [20]	Multiple	RS 01, 02, 03, 09; MA 04, 06
**8**	2020, Barry, A.et al. [15]	Multiple	VL 01-06; SE 12; RIA 20
**9**	2020, Guzman J, et al. [11]	Multiple	RS, MA, VL, MC, LI, RI, LT, CT, LR
**10**	2020, Preston C, et al. [42]	Multiple	RS, MA, VL, MC, LI, RI, LT, CT, LR
**11**	2019, Keyter A, et al. [44]	Multiple	RS 09; MA 02, 04, 06
**12**	2018, Chong, et al. [45]	Multiple	RS 09.01, RS 05, MA 01.10, MA 04.04, MC.06.03
**13**	2016, Liu P, et al. [50]	Multiple	RS 01, 02, 03, 09, 10
**14**	2014, Yao JC, et al. [54]	Multiple	RS.06.05, RS.09.02-05, RS.09.07-09.
**15**	2012, Yang S, et al. [57]	Multiple	RS 06, RS 10, MA 06; VL 05, MC 05.01, RIA.06, RIA 13, RIA 25
**16**	2022, Bujar M, et al. [26]	RS only	RS 02, 03, 04, 05, 09, 10
**17**	2022, Mahdavi M, et al. [30]	RS only	RS 06
**18**	2020, Saaristo V, et al. [43]	RS only	RS 01, RS 02, RS 05.01, RS 09, RS 10
**19**	2017, Mery G, et al. [49]	RS only	RS 07
**20**	2017, Li R, et al. [48]	RS only	RS 06, RS 09
**21**	2015, Yang S, et al. [53]	RS only	RS 04.01, RS 06-08, RS 10.02, RS 10.05
**22**	2005, Cooke J, et al. [60]	RS only	RS 06
**23**	2022, Keyter A, et al. [29]	MA only	RS 03, 05, 09, 10; MA 03, 04, 05, 06
**24**	2022, Sithole T, et al. [31]	MA only	MA 04, 06
**25**	2021, Rodier C, et al. [35]	MA only	MA 01
**26**	2021, Sithole T, et al. [37]	MA only	MA 04, 06
**27**	2020, Keyter, A., et al. [39]	MA only	MA 01-06
**28**	2020, Liberti, L., et al. [40]	MA only	MA 06
**29**	2020, Patel, P., et al. [41]	MA only	MA 06
**30**	2020, Sani, N.M., et al. [19]	MA only	MA 06
**31**	2018, Mashaki Ceyhan E, et al. [46]	MA only	MA 01, 04, 06
**32**	2018, Tang WY, et al. [47]	MA only	MA 03
**33**	2013, Liu, LL., et al. [56]	MA only	MA 04
**34**	2009, McAuslane N, et al. [58]	MA only	MA 04, 06
**35**	2007, Hirako, M.,er al. [59]	MA only	MA 06

**36**	2022, Garashi H Y, et al. [28]	VL only	VL 01, 02, 04, 05
**37**	2022, Lavery C, et al. [14]	VL only	N/A
**38**	2021,Russom M, et al. [36]	VL only	N/A
**39**	2020, Hartmann K, et al. [38]	VL only	VL 02, 04, 06
**40**	2016, Zhang MY, et al. [51]	MC only	MC.04
**41**	2015, Chen YC, et al. [52]	MC only	MC.02.01-02, MC.03.01-02, MC. 04.02-04, MC.04.07, MC.05.02, MC.06.01
**42**	2014, Zhang X, et al. [55]	MC only	RS 05.02, RS 05.08, RS 05.10, RS 07, RS 08; MC 03.01, MC 02, MC 05.
**43**	2022, Owusu Sekyere S, et al. [12]	CT only	CT 01.01, CT 01.05, CT 01.11, CT 04.07, CT 06.04

Abbreviations: RS, National Regulatory System; SE, Stakeholder engagement; RIA: Regulatory Impact Assessment; ePE: ex Post Evaluation; MA, Registration and Marketing Authorization (MA); VL, Vigilance; MC, Market Surveillance and Control; LI, Licensing Establishments; RI, Regulatory Inspection; LT: Laboratory Testing; CT: Clinical Trials Oversight; LR: NRA Lot Release.
